# International society of sports nutrition position stand: nutrient timing

**DOI:** 10.1186/s12970-017-0189-4

**Published:** 2017-08-29

**Authors:** Chad M. Kerksick, Shawn Arent, Brad J. Schoenfeld, Jeffrey R. Stout, Bill Campbell, Colin D. Wilborn, Lem Taylor, Doug Kalman, Abbie E. Smith-Ryan, Richard B. Kreider, Darryn Willoughby, Paul J. Arciero, Trisha A. VanDusseldorp, Michael J. Ormsbee, Robert Wildman, Mike Greenwood, Tim N. Ziegenfuss, Alan A. Aragon, Jose Antonio

**Affiliations:** 1 Exercise and Performance Nutrition Laboratory, School of Health Sciences, Lindenwood University, St. Charles, MO USA; 2 IFNH Center for Health & Human Performance, Department of Kinesiology & Health, Rutgers University, New Brunswick, NJ USA; 3 Health Science Department, Program of Exercise Science, CUNY Lehman College, Bronx, NY USA; 4 Institute of Exercise Physiology and Wellness, University of Central Florida, Orlando, FL USA; 5 Performance & Physique Enhancement Laboratory, Exercise Science Program, University of South Florida, Tampa, FL USA; 6 Human Performance Lab, Department of Exercise Sport Science, University of Mary Hardin-Baylor, Belton, TX USA; 7 Department of Athletics, Florida International University, Miami, FL USA; 8 Applied Physiology Laboratory, Department of Exercise and Sport Science, University of North Carolina-Chapel Hill, Chapel Hill, NC USA; 9 Exercise & Sport Nutrition Lab, Human Clinical Research Facility, Department of Health & Kinesiology, Texas A&M University, College Station, TX USA; 10 Exercise and Biochemical Nutrition Laboratory, Department of Health, Human Performance, and Recreation, Baylor University, Waco, TX USA; 11 Human Nutrition and Metabolism Laboratory, Health and Exercise Sciences Department, Skidmore College, Saratoga Springs, NY 12866 USA; 12 Department of Exercise Science and Sport Management, Kennesaw State University, Kennesaw, GA USA; 13 Department of Nutrition, Food and Exercise Sciences, Institute of Sport Sciences and Medicine, Florida State University, Tallahassee, FL USA; 14 University of KwaZulu-Natal, Biokinetics, Exercise and Leisure Studies, Durban, 4000 South Africa; 15 Post Active Nutrition, 111 Leslie St, Dallas, TX USA; 16 The Center for Applied Health Sciences, Stow, OH USA; 17 Department of Family Environmental Sciences, California State University, Northridge, CA USA; 18 Department of Health and Human Performance, Nova Southeastern University, Davie, FL USA

**Keywords:** Position stand, Exercise, Nutrition, Timing, Macronutrients, Performance, Micronutrients, Nutrients

## Abstract

The International Society of Sports Nutrition (ISSN) provides an objective and critical review regarding the timing of macronutrients in reference to healthy, exercising adults and in particular highly trained individuals on exercise performance and body composition. The following points summarize the position of the ISSN:

1. Nutrient timing incorporates the use of methodical planning and eating of whole foods, fortified foods and dietary supplements. The timing of energy intake and the ratio of certain ingested macronutrients may enhance recovery and tissue repair, augment muscle protein synthesis (MPS), and improve mood states following high-volume or intense exercise.
2. Endogenous glycogen stores are maximized by following a high-carbohydrate diet (8–12 g of carbohydrate/kg/day [g/kg/day]); moreover, these stores are depleted most by high volume exercise.
3. If rapid restoration of glycogen is required (< 4 h of recovery time) then the following strategies should be considered:
  - aggressive carbohydrate refeeding (1.2 g/kg/h) with a preference towards carbohydrate sources that have a high (> 70) glycemic index
  - the addition of caffeine (3–8 mg/kg)
  - combining carbohydrates (0.8 g/kg/h) with protein (0.2–0.4 g/kg/h)
4. Extended (> 60 min) bouts of high intensity (> 70% VO_2_max) exercise challenge fuel supply and fluid regulation, thus carbohydrate should be consumed at a rate of ~30–60 g of carbohydrate/h in a 6–8% carbohydrate-electrolyte solution (6–12 fluid ounces) every 10–15 min throughout the entire exercise bout, particularly in those exercise bouts that span beyond 70 min. When carbohydrate delivery is inadequate, adding protein may help increase performance, ameliorate muscle damage, promote euglycemia and facilitate glycogen re-synthesis.
5. Carbohydrate ingestion throughout resistance exercise (e.g., 3–6 sets of 8–12 repetition maximum [RM] using multiple exercises targeting all major muscle groups) has been shown to promote euglycemia and higher glycogen stores. Consuming carbohydrate solely or in combination with protein during resistance exercise increases muscle glycogen stores, ameliorates muscle damage, and facilitates greater acute and chronic training adaptations.
6. Meeting the total daily intake of protein, preferably with evenly spaced protein feedings (approximately every 3 h during the day), should be viewed as a primary area of emphasis for exercising individuals.
7. Ingestion of essential amino acids (EAA; approximately 10 g)either in free form or as part of a protein bolus of approximately 20–40 g has been shown to maximally stimulate muscle protein synthesis (MPS).
8. Pre- and/or post-exercise nutritional interventions (carbohydrate + protein or protein alone) may operate as an effective strategy to support increases in strength and improvements in body composition. However, the size and timing of a pre-exercise meal may impact the extent to which post-exercise protein feeding is required.
9. Post-exercise ingestion (immediately to 2-h post) of high-quality protein sources stimulates robust increases in MPS.
10. In non-exercising scenarios, changing the frequency of meals has shown limited impact on weight loss and body composition, with stronger evidence to indicate meal frequency can favorably improve appetite and satiety. More research is needed to determine the influence of combining an exercise program with altered meal frequencies on weight loss and body composition with preliminary research indicating a potential benefit.
11. Ingesting a 20–40 g protein dose (0.25–0.40 g/kg body mass/dose) of a high-quality source every three to 4 h appears to most favorably affect MPS rates when compared to other dietary patterns and is associated with improved body composition and performance outcomes.
12. Consuming casein protein (~ 30–40 g) prior to sleep can acutely increase MPS and metabolic rate throughout the night without influencing lipolysis.

## Background

The International Society of Sports Nutrition (ISSN) published the first position stand devoted to the practice of nutrient timing in 2008 [1]. Consequently, this paper has been accessed approximately 122,000 times. In the past nine years, multiple lines of research have explored questions directly related to the timing of nutrients that further refines information about evidence-based nutritional recommendations. Nutrient timing involves the purposeful ingestion of all types of nutrients at various times throughout the day to favorably impact the adaptive response to acute and chronic exercise (i.e., muscle strength and power, body composition, substrate utilization, and physical performance, etc.). Importantly, much of the interest and available research centers upon outcomes related to those who are regularly competing in some form of aerobic or anaerobic exercise; however, nutrient timing strategies may offer favorable outcomes for non-athletic and clinical populations.

From a historical perspective, nutrient timing was first conceptualized in the 1970s and 1980s with the initial work that examined the effects of increased carbohydrate feedings on glycogen status and exercise performance [2, 3]. Ivy and colleagues [4] were one of the first groups to illustrate that carbohydrate timing can influence post-exercise rates of glycogen resynthesis. While strategies surrounding carbohydrates were the first to be explored, there has been a growing body of research over the last several years that has examined the effect of protein and amino acids, with and without carbohydrates, as a nutrient timing strategy [1, 5].

Due to the volume of research investigating this concept, the need to revise and update the original document is evident. In line with the previous publication, the updated version focuses on timing considerations for two macronutrients: carbohydrates and proteins. When considering fat, research examining a specific timing question has yet to take shape. As researchers continue to explore the manipulation of fat and carbohydrate intake (e.g., ‘train low, perform high’) [6], it is possible that future recommendations may include the timing of fat intake. It is exciting to note that new research has begun to examine the impact of timed calcium (a micronutrient) intake on its ability to affect markers of bone resorption during prolonged cycling exercise [7–10] and animal models have explored the potential role of timing iron intake on various health-related outcomes [11, 12]. This research, however, is in its infancy and more studies are needed to better understand these implications. Furthermore, future versions of this position stand may need to consider expanding the document’s scope to include other ergogenic aids. For instance, research related to caffeine [13], creatine [14–16] and bicarbonate [17] have indicated that timing may affect the acute and chronic response to exercise. Therefore, the primary purpose of this updated position stand is to refine recommendations made related to the timed consumption of carbohydrates and protein and how this can potentially affect the adaptive response to exercise.

To expand upon the previous version, the current position stand now discusses research and recommendations related to meal patterns, timing, and distribution of protein, meal frequency and nighttime eating. It is the contention of the ISSN that these topics also fall under the purview of nutrient timing. Additionally, non-athletic or specialized clinical populations may also derive benefit from these strategies. Throughout each section, an attempt has been made to first highlight outcomes from acute studies before discussing those derived from training studies spanning several weeks or more.

### Carbohydrate

Moderate to high intensity (e.g., 65–80% VO_2_max) endurance activities as well as resistance-based workouts (e.g., three to four sets using ~ 6–20 repetition maximum [RM] loads) rely extensively upon carbohydrate as a fuel source; consequently, endogenous (liver: ~ 80–100 g and skeletal muscle: 300–400 g) glycogen stores are of critical importance. It is well documented that glycogen stores are limited [18, 19] and operate as a predominant source of fuel for up to a few hours during moderate to high-intensity aerobic exercise (e.g., 65–85% VO_2_max) [20, 21]. During resistance training, performing six sets of 12RM leg extension exercise has been shown to reduce glycogen stores in the *vastus lateralis* by 39% [22]. Importantly, as glycogen levels decline, the ability of an athlete to maintain exercise intensity and work output also decreases [19] while rates of tissue breakdown increase [23, 24]. The simplest guideline to maximize endogenous glycogen stores is for a high-performance athlete to ingest appropriate amounts of carbohydrate relative to their intensity and volume of training. Recommended daily intakes of carbohydrate are commonly reported to be 5–12 g/kg/day, with the upper end of this range (8–10 g/kg/day) reserved for those athletes that are training at moderate to high intensities (≥ 70% VO_2_max) upwards of 12 h per week [25–27]. In the absence of considerable muscle damage, this carbohydrate intake level has been shown to maximize glycogen storage. Percentage-based recommendations (60–70% carbohydrates of total daily caloric intake) have fallen out of favor due to their inability to appropriately prescribe required carbohydrate amounts in athletes eating high amounts of food or in those who may be following a restricted energy intake.

It should be noted that most of the recommendations for carbohydrate intake are based on the needs of endurance athletes, and in particular, male endurance athletes. Moreover, studies have indicated that trained female athletes do not oxidize fat and carbohydrate at the same rates as males and may deplete endogenous glycogen stores to different degrees [28–31]. Perhaps those involved in strength-power sports need a lower intake of carbohydrate and instead should focus more on prioritizing their carbohydrate intake in the days leading up to competition, but more research is required as this topic has been critically evaluated in a review by Escobar et al. [32]. It should be noted that athletes often fail to meet recommended amounts of energy and carbohydrate; consequently [33], strategies to replenish carbohydrate stores may take priority toprepare for maximal performance in the next competition.

#### Endurance training

The first nutrient timing strategy centered solely upon the strategic intake of carbohydrate as part of “carbohydrate loading” protocols in the days leading up to prolonged endurance competitions. Initial work by Karlsson and Saltin in the 1970s reported that a period of high-volume exercise training while consuming limited amounts of carbohydrates for three to four days followed by a diet providing > 70% carbohydrate (~ 8 to 10 g/kg/day), while sharply reducing training volume, facilitated supersaturation of muscle glycogen and an improved pace of training for more prolonged periods of time [3]. Sherman and colleagues [2, 34] also demonstrated success at maximizing intramuscular glycogen stores using similar approaches. Alternatively, Bussau et al. [35] required study participants to ingest high-glycemic carbohydrate (10 g/kg/day) for one day after completing a Wingate anaerobic capacity test which resulted in a near doubling of baseline muscle glycogen concentrations. A similar approach by Fairchild et al. [36] yielded similar results and highlights the ability to forgo a “glycogen depletion” phase and instead to simply reduce training volume for three to four days while simultaneously consuming a very high-carbohydrate diet (8–10 g/kg/day) for one to three days to maximize intramuscular glycogen levels. Overall, the ability of carbohydrate loading strategies to rapidly increase and maximize muscle glycogen levels is currently unquestioned, and many athletes and coaches are encouraged to consider making use of such a dietary regimen in the days leading up to a competitive event, particularly if their activity will significantly deplete endogenous skeletal muscle glycogen. It is important to mention that due to noted sex differences related to carbohydrate metabolism and the supercompensation of glycogen stores, female athletes may need to significantly increase total caloric intake over these “loading days” to achieve effects similar to males [31].

The hours leading up to competition are often a highly prioritized period of feeding and studies have indicated that strategic fuel consumption can help to maximize muscle and liver glycogen levels. Carbohydrate feedings during this time increase endogenous glycogen stores while also helping to maintain blood glucose levels. Notably, Coyle et al. [19] reported that consumption of a high-carbohydrate meal 4 h before 105 min of cycling exercise at 70% VO_2_max after an overnight fast significantly increased both muscle and liver glycogen while also increasing rates of carbohydrate oxidation and utilization of muscle glycogen. In addition to increasing stored glycogen, other studies have reported significant improvements in aerobic exercise performance [37–39]. However, not all studies have demonstrated a performance-enhancing effect. Nonetheless, it is commonly recommended to consume snacks or meals high in carbohydrate (1–4 g/kg/day) for several hours before higher-intensity (≥ 70% VO_2_max), longer duration (> 90 min) exercise. Additionally, and as a measure of practical importance, the need to ingest a pre-exercise meal or snacks high in carbohydrate goes up when the athlete has consumed relatively small amounts of carbohydrate in the days leading up to a competition or has not allowed for appropriate amounts of rest and recovery [20, 24].

In the final (< 4) hours before a competition, the athlete’s priority should still be to maximize or maintain optimal levels of muscle and liver glycogen. In this respect, another priority becomes maintaining a favorable balance with the digestive system and avoiding the consumption of too much food or fluid before competition. Practically speaking, many endurance events begin in the early morning hours and finding an adequate balance between rest and fuel must be considered. In this respect, two studies have reported that solid or liquid forms of carbohydrates similarly promote glycogen resynthesis allowing athletes more flexibility when selecting food sources [40, 41]. A certain degree of dogma still clouds the recommendation to ingest certain types of carbohydrate, or avoid carbohydrate altogether, in the final few hours before an event. The source of this practice stems from initial findings of Foster and colleagues [42] who reported a negative, hypoglycemic response to carbohydrate ingestion directly preceding (< 60 min) exercise. From these findings, it has been surmised that excessive carbohydrate consumption, and in particular fructose consumption, in the initial hours before exercise may negatively impact exercise performance perhaps due to rebound hypoglycemia. Indeed, given the rise in insulin due to carbohydrate ingestion coupled with up-regulation of GLUT-4 transporters from the initiated exercise stimulus, there may be a decrease, rather than increase, in blood glucose at the onset of activity that could negatively impact performance. However, while a number of athletes may be affected by this phenomenon, a study by Moseley et al. [43] demonstrated that any “rebound hypoglycemia” response is effectively negated by what would be the equivalent of a proper warm-up and that shifting carbohydrate intake closer (15 min vs. 75 min) to when the exercise bout is scheduled to begin can minimize the likelihood of these symptoms. A 1997 review by Hawley and Burke summarized the results of several studies that provided some form of carbohydrate at least 60 min before exercise. They found no adverse impact on performance. In fact, multiple studies reported performance increases of 7–20% [44]. Moreover, Galloway and colleagues [45] used a double-blind, placebo-controlled approach to compare performance outcomes related to ingestion of a placebo or a 6.4% carbohydrate beverage either 30 min or 120 min before a controlled bout of cycling at 90% peak power. Ingesting carbohydrate 30 min before exercise led to greater increases in exercise capacity. In contrast, two studies were completed by Febrraio [46, 47] that required the ingestion of high or low-glycemic carbohydrates 30–45 min before completing bouts of exercise that spanned 135–150 min at approximately 70% VO_2_max. They concluded that performance was similar for both types of carbohydrate.

The delivery of carbohydrate remains a priority once a workout or competition commences. Most research has employed study designs that integrate some form of continuous aerobic exercise, and within these studies it has been consistently demonstrated that providing carbohydrate (230–350 mL of a 6–8% carbohydrate solution) at regular intervals (every 10–12 min) can optimize performance and maintain blood glucose levels [48, 49]. Several studies have indicated that the pattern or timing of carbohydrate feedings surrounding endurance exercise may be important. For example, Fielding and colleagues [50] required cyclists to ingest the same dose of carbohydrate every 30 min or every 60 min over the course of a four-hour exercise bout. When carbohydrate was ingested more frequently, performance was improved. Two contrasting papers that operate as extensions of this work include work by Schweitzer et al. [51] who concluded that preferentially delivering carbohydrate during the first or second half of a controlled cycling exercise bout offered no enhancement of performance, while a similar study design by Heesch and colleagues [52] indicated that providing carbohydrate consistently throughout or in the latter half of a 2-h cycling exercise bout at 62% of peak power decreased the time it took to cover a prescribed distance (10-km) while cycling. It is important to realize that key differences such as the duration of the exercise bout, the nature of the performance assessment (fixed distance vs. time-to-exhaustion) and amount of carbohydrate that was delivered all differed between these studies and can help to explain the differences in outcomes being reported.

A classic paper by Widrick et al. [53] examined the impact of pre-exercise muscle glycogen status with carbohydrate feeding throughout a prolonged bout of exercise. Briefly, participants commenced a 70-km self-paced time trial with high or low muscle glycogen levels, which was then combined with either a carbohydrate (9% fructose) or placebo (non-caloric sweetener) beverage regularly (2.35 ml/kg/feeding every 10-km providing a total of 1.5 g/kg/trial) throughout the exercise bout. Increased power outputs were recorded when exercise began with high muscle glycogen levels, and even greater power was achieved when carbohydrate was frequently provided throughout the exercise protocol. A similar outcome was demonstrated by Febbraio and colleagues [54] where they required participants to complete four carbohydrate feedings and exercise conditions in conjunction with a two-hour bout of steady-state (SS) cycling exercise at 63% of their peak power, followed by a time trial using a standardized load. The four feeding conditions were: a) placebo beverage 30 min before and a 6.4% carbohydrate solution at a dosage of 2 g/kg throughout SS exercise, b) a 25.7% carbohydrate solution at a dosage of 2 g/kg 30 min before and placebo throughout SS exercise, c) a 25.7% carbohydrate solution at a dosage of 2 g/kg before and a 6.4% carbohydrate solution at a dosage of 2 g/kg throughout SS, and d) a 6.4% carbohydrate solution at a dosage of 2 g/kg throughout the SS exercise bout. As with the findings of Widrick et al., it was determined that pre-exercise strategies to support glycogen or blood glucose levels increase exercise performance when carbohydrate ingestion continued throughout the prescribed exercise bouts. Collectively, these findings somewhat prioritize carbohydrate feeding during the exercise session and could lead some to argue that if pre-exercise carbohydrate feeding strategies are neglected, then delivering appropriate carbohydrate throughout an exercise bout may help offset the potential for performance decrement. However, one must cautiously explore this approach as to avoid overwhelming the gastrointestinal system potentially leading to cramping and discomfort once exercise begins. In this respect one should consider the findings of Newell et al. [55] who had 20 well-trained, experienced cyclists perform four different feeding conditions (no carbohydrate [0 g/h] control, 20 g/h, 39 g/h or 64 g/h) throughout completion of a two-hour cycling bout at 95% lactate threshold (185 ± 25 watts) followed by completion of a standardized time trial. When carbohydrates were ingested at a dosage of 39 or 64 g/h, time trial performance was significantly improved compared to the control group. Importantly, no differences in performance were found between these two feeding strategies suggesting that for those athletes who may not be able to tolerate higher doses of carbohydrates, a moderate regimen of carbohydrate feeding throughout a prolonged bout of exercise can still promote similar improvements in performance. Other important considerations related to the potential ergogenic impact of carbohydrates have been critically highlighted in recent reviews by Colombani et al. [56] and later by Pochmuller et al. [57]. In both papers, the authors contend that the ability of carbohydrate administration during bouts of exercise spanning less than 70 min to operate in an ergogenic fashion is largely mixed in the literature. It was further suggested that not until exercise durations meet or exceed 90 min does the administration of a ~ 6–8% carbohydrate solution exert a consistent ergogenic benefit particularly when exercise is commenced in a fed state as opposed to the fasted state that is so often studied in this body of literature.

Whether or not these results translate to intermittent sports remains to be thoroughly investigated. A 2011 review by Phillips and colleagues [58] supports the notion that carbohydrate administration throughout intermittent, team-sport activities improves certain types of performance as well as general indicators of mental drive and acuity, but evidence regarding benefits of acute deviations in timing is still lacking. Clarke and colleagues [59] tested the hypothesis that ingesting isovolumetric amounts of a carbohydrate-electrolyte solution either in two large volumes (7 mL/kg at 0 and 45 min of exercise) or more frequent (every 15 min over the entire course of a 75-min exercise bout) feedings of smaller volumes to achieve the same total dose can favorably impact metabolic responses. No performance or capacity measurements were made, but the authors did report that either feeding pattern was able to maintain glucose, insulin, glycerol, non-esterified fatty acid, and epinephrine levels. More recently, Mizuno and colleagues [60] concluded that timing the intake of a carbohydrate gel (1.0 g/kg) did not impact the inflammatory response or exercise performance throughout completion of two 45-min bouts of intermittent (4–16 km/h) running.

The recovery of lost muscle glycogen operates as a key nutritional goal, and post-exercise ingestion of carbohydrate continues to be a popular and efficient nutrient timing strategy to maximize replenishment of lost muscle glycogen. In what is known as potentially the first study to examine an actual nutrient timing question, Ivy and colleagues [61] showed that restoration of muscle glycogen was 50% faster and more complete over a four-hour post-exercise period when a carbohydrate bolus (2 g/kg of a 25% carbohydrate solution) was delivered within 30 min versus waiting until two hours after completion of a cycling exercise bout (70 min at 68% VO_2_max followed 6 × 2-min intervals at 88% VO_2_max). Subsequent work has since refined conclusions surrounding this topic, namely that the timing of post-exercise carbohydrate administration holds the highest level of importance under two primary situations: 1) when rapid restoration of muscle glycogen is a primary goal and 2) when inadequate amounts of carbohydrate are being delivered. In light of these considerations, muscle glycogen levels can be rapidly and maximally restored using an aggressive post-exercise feeding regimen of carbohydrates. Ingesting 0.6 to 1.0 g/kg body mass within the first 30 min of completing a glycogen depleting exercise bout and again every two hours for the next four to six hours [62, 63], has been shown to promote maximal glycogen replenishment. Similarly, favorable outcomes have also been shown when 1.2 g/kg of carbohydrate were ingested every 30 min over a 3.5-h period [27, 64].

Outside of situations where rapid recovery is truly needed, and daily carbohydrate intake is matching energy demands, the importance of timed carbohydrate ingestion is notably decreased. However, in no situation has timed carbohydrate ingestion been shown to negatively impact performance or recovery. If an athlete participating in heavy exercise is not able, or even not sure if they will be able to appropriately consume the required amounts of carbohydrate throughout the day then the strategically timed ingestion of carbohydrate may accelerate muscle glycogen re-synthesis. When prolonged endurance exercise is completed, carbohydrate ingestion may also help promote a favorable hormonal environment [65, 66]. Finally, studies in elite athletes undergoing high volumes of training have shown that maximal glycogen levels are restored within 24 h if a diet contains ≥8 g/kg/day, and only moderate levels of muscle damage are present [41]. In support, Nicholas and colleagues [67] concluded that a daily carbohydrate intake of 9–10 g/kg/day in six trained men participating in soccer, rugby, hockey, or basketball, sufficiently replenished muscle glycogen following consecutive days of prolonged (85–90 min), intense, interval exercise.

#### Resistance training

Studies employing resistance exercise that examined some aspect of carbohydrate timing are limited. Multiple studies have demonstrated that resistance exercise can significantly decrease muscle glycogen concentration [22, 68–70], though these decreases are modest in comparison to exhaustive endurance exercise. However, the provision of pre-exercise carbohydrate to individuals performing resistance-style exercise in a moderately glycogen depleted state may not have an ergogenic effect. To date, one study has indicated that carbohydrate administration before and during bouts of resistance exercise can improve performance, but these ergogenic outcomes were only seen in the second session of resistance exercise performed on the same day [71]. In contrast, multiple studies have failed to report an improvement in resistance exercise performance [72–74]. One study involving pre-exercise and during exercise delivery of carbohydrate throughout a bout of resistance exercise has been shown to minimize the loss of muscle glycogen. Briefly, study participants were given a carbohydrate dose of 1.0 g/kg pre-workout and a 0.5 g/kg carbohydrate every 10 min throughout a 40-min resistance exercise bout and found that muscle glycogen losses were reduced by 49% when compared to glycogen changes with ingestion of a placebo drink; however, isokinetic muscle performance was not influenced [73].

In reviewing all of the timing considerations related to carbohydrate intake, strategies to maximize muscle and liver glycogen levels should first consist of following a brief period of reduced training volume in conjunction with a high daily intake of carbohydrate (≥ 8 g/kg/day). In the hours leading up to competition, glycogen levels are best maintained or increased by consuming high carbohydrate (1–4 g/kg/day) meals or snacks for several hours before commencement of training or competition. Athletes are encouraged to continue consuming small amounts of a carbohydrate solution or small snacks (bars, gels, etc.) to maintain liver glycogen levels and to help prevent hypoglycemia. Ingestion of carbohydrate during endurance type exercise maintains blood glucose levels, spares glycogen [75], and will likely enhance performance. Post-exercise consumption of carbohydrate is necessary and in situations where minimal recovery time is available, aggressive carbohydrate feeding is recommended. Although preliminary, initial work in intermittent, high-intensity activities suggest that carbohydrate timing may support metabolic outcomes, while performance results remain mixed, as do studies involving resistance exercise. For further inquiry, excellent reviews on the topic of carbohydrate and performance are available [20, 21, 48, 49, 76].

### Carbohydrate + protein

#### Endurance training

Carbohydrate + protein combinations are a traditional strategy employed by endurance as well as strength-power athletes to increase exercise performance, promote glycogen repletion, minimize muscle damage, and promote a positive nitrogen balance. A small number of studies have examined pre-endurance exercise ingestion of carbohydrate + protein on performance as well as metabolic outcomes, but very few have directly investigated the impact of altering the timing of when nutrients were administered. Ivy and colleagues [77] recruited trained cyclists to complete a three-hour bout of cycling exercise at an intensity of 45–75% VO_2_max before exercising to exhaustion at 85% VO_2_max. In a crossover fashion, participants ingested either a 7.75% carbohydrate or a 7.75% carbohydrate + 1.94% protein solution. When protein was added to carbohydrate, endurance was significantly improved. In a similar fashion, Saunders and colleagues [78] had participants cycle to exhaustion on two separate occasions (75–85% VO_2_max) within 24 h while ingesting a carbohydrate or a carbohydrate + protein solution throughout the exercise bout (1.8 mL/kg every 15 min) followed by a single bolus dose (10 mL/kg) immediately after exhaustion. The carbohydrate + protein combination resulted in significantly improved performance as well as a reduction in muscle damage. The same research group [79] used a nutrient gel and again reported that ingestion of a carbohydrate (0.146 g/kg) + protein (0.0365 g/kg) combination throughout an exhaustive bout of cycling exercise significantly improved cycling performance. While none of these studies directly examined a timing comparison, they all demonstrate that pre-exercise administration of carbohydrate + protein combinations can favorably impact endurance performance. Furthermore, the addition of protein (to carbohydrate) has been shown to increase the speed of glycogen recovery when a short recovery window is available or if sub-optimal amounts of carbohydrate have been delivered and can also help to reduce symptoms of muscle damage [80]. Notably, no studies have demonstrated that addition of protein to carbohydrate to a pre-exercise feeding in these amounts may hinder exercise performance. Similarly, Rustad and colleagues [81] reported that adding protein (0.4 g/kg/h) to carbohydrate (0.8 g/kg/h) within 2 h of completing an initial exhaustive bout of cycling exercise led to a significant increase in cycling performance the next morning when compared to ingesting just carbohydrate alone, thus suggesting improved recovery.

To support recovery upon completion of exercise bouts that can deplete stored fuels and may cause significant damage to the muscle tissue, post-exercise nutrient timing strategies are of great interest. Ivy et al. [82] required cyclists to complete a 2.5-h bout of cycling (65–75% VO_2_max) before consuming a combination of carbohydrate + protein (80 g carbohydrate + 28 g protein + 6 g fat) or two different doses (High: 108 g of carbohydrate + 6 g fat or Low: 80 g carbohydrate + 6 g fat) of carbohydrate immediately after and 2 h after completing the exercise session. While timing was not specifically investigated, the carbohydrate + protein combination led to greater glycogen recovery during the four-hour investigative window employed by the research team. These findings replicated previous findings [83] by this research group and led them to conclude that the addition of protein favorably promoted early phases of glycogen recovery. Berardi et al. later published two similar studies [84, 85] that also showed that the provision of a combination of carbohydrate + protein facilitated greater recovery of muscle glycogen when ingested soon after the completion of a workout and before a subsequent endurance exercise bout.

As more research has been completed on the topic, the potential benefits of adding protein have been questioned. For example, Jentjens and colleagues [63] failed to show an improvement in muscle glycogen restoration with a combination of carbohydrate (1.2 g/kg/h) + protein (0.4 g/kg/h) in comparison to ingesting only the carbohydrate dose over a three-hour recovery period. Howarth and colleagues [86] later came to a similar conclusion regarding the addition of protein and extended these findings also to report that a higher dose of carbohydrate (1.6 g/kg/h) did not further promote glycogen resynthesis. Thus, it appears that protein addition augments glycogen recovery when carbohydrate ingestion is < 1.2 g/kg/h.

#### Resistance exercise

A small number of studies are available that examined the effect of ingesting carbohydrate + protein before resistance exercise. For example, Kraemer and colleagues [87] had participants ingest a combination of carbohydrate, protein, and fat or an isoenergetic maltodextrin placebo for seven days before two consecutive days of resistance exercise. On both occasions, the supplement was ingested 30 min before beginning the exercise bout, and the multi-nutrient supplement significantly improved vertical jump power and the number of repetitions performed at 80% 1RM. A similar outcome was reported by Baty and colleagues [88] where they had 34 males complete an acute bout of heavy resistance training (3 sets × 8 reps @ 90% 1RM) while consuming either a carbohydrate (6.2% carbohydrate) or a carbohydrate + protein (6.2% carbohydrate + 1.5% protein) solution before, during, and after the exercise bout. While performance was not affected, significantly greater levels of insulin and lower levels of cortisol were found when the carbohydrate + protein combination was ingested. Moreover, markers of muscle damage (e.g., myoglobin and creatine kinase) were reduced throughout the first 24 h of recovery when the carbohydrate + protein combination was consumed. These two studies provided a combination of carbohydrate + protein at some point before the resistance exercise sessions, however these studies were not designed to examine whether pre-exercise feeding of carbohydrate + protein was responsible for improved exercise performance or adaptations.

Tipton and colleagues [89] completed one of the first studies to directly examine whether the timing of carbohydrates + EAA altered MPS rates. In this investigation, research participants completed a single bout of lower-body resistance exercise while ingesting the same combination of carbohydrate (35 g of sucrose) + 6 g EAA either immediately before or immediately after completion of the exercise bout. Nutrient ingestion immediately before the exercise bout increased MPS significantly more than when the carbohydrate + EAA combination was consumed after the resistance exercise session. A few years later, however, Fujita and colleagues [90] attempted to replicate their study findings and instead determined that MPS rates were similar between pre-exercise and post-exercise ingestion. While many people use the Fujita paper to discount the pre-exercise period, it should be noted that significant increases in MPS rates occurred when nutrients were administered before and after the resistance training bout in comparison to a non-energetic control suggesting that nutrient delivery itself*,* as opposed to *timing* of delivery, should be a larger priority. White and colleagues [91] conducted a study to specifically examine if timed ingestion of carbohydrate + protein timing influenced force production and markers of muscle damage. For this study, 27 adult participants ingested either a non-caloric sweetener or a carbohydrate (75 g) + protein (23 g) combination 15 min before or 15 min after a bout of damaging resistance exercise and found that neither the nutrients themselves, nor their timing, influenced changes in force production or blood levels of muscle damage markers. The results suggest that MPS rates can be acutely increased if a combination of carbohydrate + protein is consumed either before or after, but changes in force production or muscle damage may not be impacted by timed ingestion of a carbohydrate + protein combination.

The acute effect of ingesting a carbohydrate + protein or EAA combination throughout resistance exercise has been studied [92–96]; however, as with other time periods, no studies have truly examined the question of timing. In this respect, a series of studies published by Bird and colleagues [93–96] has investigated the influence of consuming either carbohydrate or carbohydrate + EAA on measures of acute performance, hormonal responses and circulating levels of blood proteins associated with muscle damage. In the first study, 32 participants were randomized to ingest either a 6% carbohydrate solution, a 6% carbohydrate solution + 6 g of EAA or a non-nutritive placebo regularly throughout a 60-min bout of resistance training. Findings from this study indicated that serum cortisol levels were reduced when either a 6% carbohydrate solution or a 6% carbohydrate + 6 g EAA solution were ingested in comparison to a non-energetic placebo [94]. A later publication from this investigation reported that urinary muscle protein breakdown markers were reduced by 27% when the carbohydrate + EAA combination was consumed while the placebo group experienced a 56% increase [95].

A later study by Bird et al. [93] used a ‘triphasic’ approach where they delivered a combination of carbohydrate + amino acids before, during and after a single bout of resistance exercise. Using a crossover study design, participants also ingested a placebo that consisted of water flavored with a non-nutritive sweetener in similar volumes at the same times. They reported that delivering nutrients (versus none at all) did significantly increase the volume of exercise completed and reduced concentrations of serum proteins indicative of muscle damage. Along these lines, Beelen and colleagues [92] also completed an acute study design that required study participants to ingest in a fed state a carbohydrate + hydrolyzed casein protein combination at a dose of 0.15 g/kg body mass before initiating a two-hour resistance-training session and at 15-min intervals throughout the bout. Compared to placebo, the carbohydrate + protein combination significantly lowered rates of protein breakdown and increased fractional synthetic rates of muscle proteins by 49 ± 22%, resulting in a five-fold increase in protein balance.

Chronic studies examining carbohydrate + protein ingestion with resistance training have also been conducted. Bird et al. [96] examined the impact of consuming a 6% carbohydrate +6 g EAA solution throughout bouts (two bouts per week) of resistance exercise over a 12-week period. Urinary concentrations of 3-methyl-histidine were reduced by 26% when the carbohydrate + EAA combination was ingested, which was significantly different from the 52% increase observed in the placebo group. Also, the cross-sectional areas of type I, IIa, and IIb muscle fibers increased in comparison to the changes seen when solutions containing either just carbohydrate (6%) or EAA (6 g) were ingested. While these findings are encouraging, the studies are limited by the dosage of EAA provided as other studies have indicated that higher EAA doses (up to 12 g) may maximally stimulate MPS. As such, future research in this area should identify if different doses of EAA or combining a carbohydrate solution with varying doses of intact proteins consumed during resistance exercise bouts can further impact performance and resistance training adaptations. In this respect, when sufficient protein is supplied, it may be that carbohydrate has no additional adaptive benefit. As an example of this, Hulmi and colleagues [97] showed no benefit in resistance training adaptations when a combination of maltodextrin carbohydrate (34.5 g) + whey protein concentrate (37.5 g) was ingested immediately following each workout of a regimented 12-week resistance training protocol as compared to consuming the protein supplement alone. Cribb and Hayes [16] randomized trained male participants to ingest identical amounts of carbohydrate + protein + creatine either immediately before and immediately after resistance training or in the morning and evening during a 10-week resistance-training program. Changes in strength, hypertrophy, and body composition were assessed, and significant increases in lean body mass, 1RM strength, type II muscle fiber cross-sectional area, and higher muscle creatine and glycogen levels were found when the supplements were consumed immediately before and after workouts as opposed to consuming them in the morning and evening. While seemingly different than the results of Hulmi, these results indicate that close temporal ingestion of a combination of carbohydrate + protein + creatine may afford favorable outcomes relative to resistance training adaptations and does not necessarily state that a carbohydrate + protein combination is better than simply ingesting similar amounts of protein. Furthermore, Cribb and Hayes also provided creatine while the other studies did not, which has been shown in multiple investigative scenarios to augment the muscular adaptations seen while resistance training [98–100].

Carbohydrate + protein combinations while resistance training are suggested to augment muscle development via an increased insulin response. Specifically, insulin promotes anti-catabolic effects in muscle [101], thereby shifting protein balance to favor anabolism. However, insulin-mediated effects on reducing proteolysis plateau within a range of ~ 15–30 μIU/mL [102, 103], and these levels are achieved by consuming a 45 g bolus of whey protein isolate alone [104]. This would suggest that post-workout carbohydrate supplementation likely exerts minimal influence from a muscle development standpoint provided adequate protein is consumed. Towards this end, Staples and colleagues [105] compared the impact of a carbohydrate (50 g maltodextrin) + protein (25 g whey protein) combination on rates of MPS observed after completing a single bout of lower-body resistance exercise. The authors reported that the carbohydrate + protein combination failed to further stimulate increases in MPSwhen compared to ingesting only protein. Furthermore, Rasmussen and colleagues [106] found no difference in amino acid balance when 35 g of sucrose + 6 g of EAA were ingested either 1 h or 3 h after completion of a bout of resistance training.

In summary, ingestion of carbohydrate + protein (or amino acids) in close temporal proximity to or throughout both endurance and resistance exercise may operate as an effective strategy to favorably affect performance of a subsequent exercise bout as well as adaptations from regular bouts of training. Towards this end, enhancements in endurance performance, as well as improved recovery of reduced muscle glycogen have also been consistently reported when carbohydrate + protein combinations have been consumed surrounding exercise bouts, particularly if lower quantities of carbohydrate are consumed. However, when optimal carbohydrate is delivered the impact of adding protein (irrespective of when it is provided) appears to offer little to no additional benefit on endurance or resistance exercise performance as well as the recovery of reduced muscle glycogen. Much like the work on glycogen recovery, studies involving resistance training and optimization of adaptations seen from resistance training also point towards a higher priority being given towards the total amount of protein consumed during the day. Therefore, if total protein needs are met, the importance of adding carbohydrate (and even more so in a timed fashion) may be limited. A key point of discussion, however, lies with whether or not total energy needs are also being met, particularly in athletes undergoing large volumes of training and more so in those athletes that have high amounts of lean as well as body mass. In these situations, it certainly remains possible that the addition of carbohydrate to a protein feeding may help the athlete achieve an appropriate energy intake, which certainly may go on to impact the extent to which adaptations occur. For athletes who are likely combining resistance training sessions with sport-specific training, the provision of carbohydrate + protein in close proximity to each session would be warranted in order to optimize recovery for subsequent bouts and adaptation.

### Protein

#### Endurance training

The role of amino acids and/or protein consumption with regards to endurance exercise is not well known. Pasiakos and colleagues [107] had cyclists complete two different bouts of exercise (60 min at 60% VO_2_peak) while ingesting a solution containing 10 g of the EAA with varying levels (1.87 or 3.5 g) of leucine. In response to EAA ingestion and independent of leucine content, MPS rates and several signaling proteins related to muscle hypertrophy (i.e., Akt, mTOR, p70s6k, etc.) were significantly increased. While more research certainly needs to be conducted to better identify the potential impact and role of protein intake before endurance exercise, the priority for an endurance athlete in the hours leading up to competition should be focused on appropriate carbohydrate intake to fully maximize endogenous production of glycogen.

#### Resistance training

As with endurance exercise, the majority of studies that have employed some form of protein or amino acid ingestion before bouts of resistance exercise have done so in conjunction with an identical dose during the post-exercise period as well. For example, Tipton and colleagues [108] used an acute resistance exercise and feeding model to report that MPS rates were similar when a 20-g dose of whey protein was ingested immediately before or immediately after a bout of lower body resistance training. Andersen et al. [109] were one of the first to examine the effects of ingesting protein immediately before and immediately after resistance exercise over multiple weeks. In this study, participants were randomized to ingest either 25 g of a protein blend (16.6 g whey, 2.8 g casein, 2.8 g egg white, 2.8 g glutamine) or maltodextrin immediately before and immediately after each workout over the course of 14 weeks. In the group that consumed the protein-amino acid blend, type I and type II muscle fibers experienced a significant increase in size. Also, the protein-amino acid group experienced a significant increase in squat jump height while no changes occurred in the carbohydrate group. Using a similar study design, Hoffman and colleagues [110] had collegiate football players who had been regularly performing resistance-training ingest 42 g of hydrolyzed collagen protein either immediately before and immediately after exercise, or in the morning and evening over the course of ten weeks of resistance training. In this study, the timing of protein intake did not impact changes in strength, power and body composition experienced from the resistance-training program.

When examining the discrepant findings, one must consider a few things. First, the protein source in the Hoffman et al. study was mostly a collagen hydrolysate (i.e., not the highest quality protein source); moreover, changes in body composition were determined by dual-energy x-ray absorptiometry (DEXA), which does not have the same sensitivity to identify subtle hypertrophic alterations [111] as the histochemical approaches employed by Andersen et al. [109]. Finally, the study participants in the Andersen et al. study were consuming approximately 20% more calories per day (~ 36.6 kcals/kg/day) than the participants in the Hoffman study (who consumed only 30.4 kcals/kg/day) which offers some level of explanation for the different outcomes reported in these two studies. More recently, Schoenfeld and colleagues [112] published the first longitudinal study to directly compare the effects of ingesting 25 g of whey protein isolate either immediately before or immediately after each workout. For this study, 21 resistance-trained men (> 1-year experience) followed a 10-week, three day per week whole-body heavy resistance training program (3 sets of 8 – 12RM) and concluded there were no differences in muscle mass or strength changes when the dose of whey protein was consumed pre- or post-training. This study is significant as it is the first investigation to attempt to compare pre versus post-workout ingestion of protein. The authors raised the question that the size, composition, and timing of a pre-exercise meal may impact the extent to which adaptations are seen in these studies. However, a key limitation of this investigation is the very limited training volumes these subjects performed. The total training sessions over the 10-week treatment period was 30 sessions (i.e., total of 30 h assuming each session lasted 1 h). One would speculate that the individuals who would most likely benefit from peri-workout nutrition are those who train at much higher volumes. For instance, American collegiate athletes per NCAA regulations (NCAA Bylaw 2.14) are limited to a maximum of 4 h per day and a 20-h training schedule per week [113]. Thus, the average college athlete trains more in two weeks than most subjects train during an entire treatment period in studies in this category.

In one of the only studies to use older participants, Candow and colleagues [15] assigned 38 men between the ages of 59–76 years to ingest a 0.3 g/kg protein dose before or after each workout over the course of a 12-week resistance training program. While protein administration did favorably improve resistance-training adaptations, the timing of protein (before or after workouts) did not invoke any differential change. An important point to consider with the results of this study is the sub-optimal dose of protein (approximately 26 g of whey protein) versus the known anabolic resistance that has been demonstrated in the skeletal muscle of elderly individuals [114]. In this respect, the anabolic stimulus from a 26-g dose of whey protein may not have sufficiently stimulated muscle protein synthesis or have been of appropriate magnitude to induce differences between conditions. Clearly, more research is needed to determine if a greater dose of protein delivered before or after a workout may exert an impact on adaptations seen during resistance training in an elderly population.

Limited studies are available that have examined the effect of providing protein throughout an acute bout of resistance exercise, particularly studies designed to explicitly determine if protein administration during exercise was more favorable than other times of administration. As discussed previously as part of the carbohydrate + protein section, research by Bird and colleagues [94, 95] had participants ingest a 6-g solution of EAA throughout a bout of resistance exercise and reported increases in post-exercise insulin levels and reductions in urinary levels of 3-methyl-histidine and serum levels of cortisol. However, when examined over the course of 12 weeks, the increases in fiber size seen after ingesting a solution containing 6 g of EAA alone was less than when it was combined with carbohydrate [96].

The post-exercise time period has been aggressively studied for its ability to heighten various training outcomes. While a large number of acute exercise and nutrient administration studies have provided multiple mechanistic explanations for why post-exercise feeding may be advantageous [115–119], other studies suggest this study model may not be directly reflective of adaptations seen over the course of several weeks or months [120]. As highlighted throughout the pre-exercise protein timing section, the majority of studies that have examined some aspect of post-exercise protein timing have done so while also administering an identical dose of protein immediately before each workout [16, 109, 110, 121]. Of these studies, protein [109] or carbohydrate + protein [16] consumption immediately before and immediately after resistance exercise has been shown to positively affect resistance training adaptations. These results, however, are not universal as Hoffman et al. [110] reported no impact of timing when 42 g of hydrolyzed collagen protein was ingested before and after several weeks of resistance timing. Of note, participants in the Hoffman study were all highly-trained collegiate athletes who reported consuming a hypoenergetic diet. Candow et al. [15] reported that sub-optimal doses of whey protein ingestion (0.3 g/kg, ~ 26 g) in elderly males (59–76 years) before or after resistance training workouts exerted no impact on strength and body composition changes. As mentioned previously, it is possible that the dose of protein may not have been an appropriate amount to properly stimulate anabolism.

In this respect, a small number of studies have examined the impact of solely ingesting protein after exercise. As discussed earlier, Tipton and colleagues [108] used an acute model to determine changes in MPS rates when a 20-g bolus of whey protein was ingested immediately before or immediately after a single bout of lower-body resistance training. MPS rates were significantly, and similarly, increased under both conditions. Until recently, the only study that examined the effects of post-exercise protein timing in a longitudinal manner was the 2001 work of Esmarck et al. [122]. In this study, 13 elderly men (average age of 74 years) consumed a small combination of carbohydrates (7 g), protein (10 g) and fat (3 g) either immediately (within 30 min) or 2 h after each bout of resistance exercise done three times per week for 12 weeks. Changes in strength and muscle size were measured, and it was concluded that ingesting nutrients immediately after each workout led to greater improvements in strength and muscle cross-sectional area than when the same nutrients were ingested 2 h later. While interesting, the inability of the group that delayed supplementation but still completed the resistance training program to experience any measurable increase in muscle cross-sectional area has led some to question the outcomes resulting from this study [5, 123]. Further and as discussed previously with the results of Candow et al. [15], the dose of protein (10 g) was likely an inadequate dose for a population of this age. Schoenfeld and colleagues [124] published results that directly examined the impact of ingesting 25 g of whey protein immediately before or immediately after bouts of resistance-training. All study participants trained three times each week targeting all major muscle groups over a 10-week period, and the authors concluded no differences in strength and hypertrophy were seen between the two protein ingestion groups. These findings lend support to the hypothesis that ingestion of whey protein immediately before or immediately after workouts can promote improvements in strength and hypertrophy, but the time upon which nutrients are ingested does not necessarily trump other feeding strategies.

Reviews by Aragon and Schoenfeld [125] and Schoenfeld et al. [126] critically examined the efficacy surrounding post-exercise protein administration. The authors suggested that when recommended levels of protein are consumed, the effect of timing appears to be, at best, minimal. Indeed, research shows that muscles remain sensitized to protein ingestion for at least 24 h following a resistance training bout [127] leading the authors to suggest that the timing, size and composition of any feeding episode before a workout may exert some level of impact on the resulting adaptations. In addition to these considerations, recent work by MacNaughton and colleagues [128] reported that the acute ingestion of a 40-g dose (versus 20-g) of whey protein resulted in significantly greater increases in MPS in young subjects who completed an intense, high volume bout of resistance exercise that targeted all major muscle groups. While seemingly a protein dose question (and not necessarily a timing question per se), these findings are significant from a timing perspective as the extent to which this higher dose interacts with the muscle’s ability to respond to a subsequent dose of amino acid or protein (alone or as a mixed meal) feedings remains undetermined. Notwithstanding these conclusions, the number of studies that have truly examined a timing question is rather scant. Moreover, recommendations must capture the needs of a wide range of individuals, and to this point, a very small number of studies have examined the impact of nutrient timing using highly trained athletes. From a practical standpoint, some athletes may struggle, particularly those with high body masses, to consume enough protein to meet their required daily needs. Therefore, due to the known sensitization that occurs in skeletal muscle to protein ingestion for ~ 24 h, the pragmatic recommendation is for an athlete to feed as soon as possible after a workout. In this respect, not eating does not offer any benefit regarding skeletal muscle hypertrophy and recovery from endurance and/or strength-power exercise.

### Timing and distribution of meals - time of day considerations

Evidence has surfaced that suggests what part of the day the majority of calories are consumed may affect one’s health, weight loss or body composition changes. As a starting point, it is important to highlight that most of the available research on this topic has largely used non-athletic, untrained populations except two recent publications using trained men and women [129, 130]. Whether or not these findings apply to highly trained, athletic populations remains to be seen. Keim and colleagues [131] required study participants to complete two six-week diet periods that delivered similar calories (~ 1950 kcals) and a similar macronutrient composition. In one scenario, the participants were required to consume 70% of their prescribed dietary intake during the morning meal, while in the other study group participants were required to consume 70% of their prescribed dietary intake with their evening meals. Changes in weight loss and body composition were compared, and slightly greater weight loss occurred when the majority of calories was consumed in the morning. As a caveat to what is seemingly greater weight loss when more calories are shifted to the morning meals, higher amounts of fat-free mass were lost as well, leading to questions surrounding the long-term efficacy of this strategy regarding weight management and metabolic activity. Notably, this last point speaks to the importance of evenly spreading out calories across the day and avoiding extended periods of time where no food, protein in particular, is consumed. A large observational study [132] examined the food intake of 867 free-living individuals (375 males and 492 females),and a follow-up study from the same study cohort [133] reported that the timing of food consumption (earlier vs. later in the day) was correlated to the total daily caloric intake. These findings indicate that consuming a greater proportion of one’s total daily calories earlier in the day was associated with lower daily caloric intake while shifting more of the daily caloric consumption to evening meals increased one’s total caloric intake. Indeed, one must cautiously interpret these results as they are not offering any insight into how these eating patterns may influence body composition changes or even loss of body mass, but nonetheless, provide interesting initial data on how “when” certain foods are consumed may impact total daily caloric intake.

Wu and colleagues [134] reported that meals later in the day lead to increased rates of lipogenesis and adipose tissue accumulation in an animal model and, while limited, human research has also provided support. Previously it has been shown that people who skip breakfast display a delayed activation of lipolysis along with an increase in adipose tissue production [135, 136]. More recently, Jakubowicz and colleagues [137] had overweight and obese women consume 1400 cal each day for a 12-week period. A portion of the study participants consumed 50% of their daily calories (700 kcals) during breakfast, 35% during lunch (500 kcals) and 15% during dinner (200 kcals), while the other portion of study participants consumed the exact opposite distribution 15% for breakfast (200 kcals), 35% for lunch (500 kcals) and 50% for dinner (700 kcals). Approximately 2.5 times more weight was lost, and significantly greater changes in waist circumference and body mass index values were observed, when the majority of calories were consumed at breakfast. Also, triglyceride levels decreased by 34%, greater improvements in glucose and insulin were observed, and feelings of satiety were improved in the group that consumed the majority of their calories at breakfast [137]. While these results provide insight into how calories could be more optimally distributed throughout the day, a key perspective is that these studies were performed in sedentary populations without any form of exercise intervention. Thus, their relevance to athletes or highly active populations might be limited. Furthermore, the current research approach has failed to explore the influence of more evenly distributed meal patterns throughout the day.

### Meal frequency

Meal frequency is commonly defined as the number of feeding episodes that take place each day. For years, recommendations have indicated that increasing meal frequency may serve as an effective way to influence weight loss, weight maintenance, and body composition. These assertions were based upon the epidemiological work of Fabry and colleagues [138, 139] who reported that mean skinfold thickness was inversely related to the frequency of meals. One of these studies involved 379 overweight individuals between 60 and 64 years of age while the other investigation involved 80 participants between the ages of 30–50 years of age. An even larger study published by Metzner and colleagues [140] reported that in a sample of 2000 men and women between 35 and 60 years of age, meal frequency and adiposity were inversely related. While intriguing, the observational nature of these studies does not agree with more controlled experiments. For example, a 2005 study by Farshchi et al. [141] required individuals over a 14-day period to consume either a regular, consistent pattern of six daily meals or eat anywhere from three to nine meals per day. The irregular meal pattern was found to result in increased levels of appetite, and hunger leading one to question if the energy provided in each meal was inadequate or if the energy content of each meal could have been better matched to limit these feelings while still promoting weight loss. Furthermore, Cameron and investigators [142] published what is one of the first studies to directly compare a greater meal frequency to a lower frequency. In this study, 16 obese men and women reduced their energy intake by 700 kcals per day and were assigned to one of two isocaloric groups: one group was instructed to consume six meals per day (three traditional meals and three snacks), while the other group was instructed to consume three meals per day for an eight-week period. Changes in body mass, obesity indices, appetite, and ghrelin were measured at the end of the eight-week study, and no significant differences in any of the measured endpoints were found between conditions. These results also align with more recent results by Alencar [143] who compared the impact of consuming isocaloric diets consisting of two meals per day or six meals per day for 14 days in overweight women on weight loss, body composition, serum hormones (ghrelin, insulin), and metabolic (glucose) markers. No differences between groups in any of the measured outcomes were observed. A review by Kulovitz et al. [144] concluded that when total energy intake is controlled, and when caloric restriction is employed, the influence of meal frequency on weight loss and improving one’s body composition is secondary to the total daily caloric intake. Similar conclusions were drawn in a meta-analysis by Schoenfeld and colleagues [145] that examined the impact of meal frequency on weight loss and body composition. Although initial results suggested a potential advantage for higher meal frequencies on body composition, sub-analysis indicated that findings were confounded by a single study, casting doubt as to whether the strategy confers any beneficial effects. However, it is important to note that this “outlier” study was the only one to include an exercise regimen and only lasted for two weeks. From this, one might conclude that greater meal frequency may, indeed, favorably influence weight loss and body composition changes *if* used in combination with an exercise program for a short period of time. Certainly, more research is needed in this area, particularly studies that manipulate meal frequency in combination with an exercise program in non-athletic as well as athletic populations. Finally, other endpoints related to meal frequency (i.e., glucose/insulin homeostasis, hunger and appetite levels, energy levels, etc.) may be of interest to different populations, but they extend beyond the scope of this position stand. The interested reader is referred to the ISSN’s position stand on meal frequency [146].

### Timing and distribution of protein feeding

An extension of altering the patterns or frequency of when meals are consumed is to examine the pattern upon which protein feedings occur. Researchers have clearly illustrated that upon ingestion of a meal containing protein and/or amino acids, serum levels of amino acids as well as MPS rates will rise and stay elevated for three to 5 h depending on bolus size [147, 148]. Moore and colleagues [149] examined the differences in protein turnover and synthesis rates when participants ingested different patterns, in a randomized order, of an 80-g total dose of protein over a 12-h measurement period following a bout of lower body resistance exercise. One of the protein feeding patterns required participants to consume two 40-g doses of whey protein isolate approximately 6 h apart. Another condition required the consumption of four, 20-g doses of whey protein isolate every 3 h. The final condition required the participants to consume eight, 10-g doses of whey protein isolate every 90 min. Rates of muscle protein turnover, synthesis, and breakdown were compared, and the authors concluded that protein turnover and synthesis rates were greatest when intermediate-sized (20-g) doses of whey protein isolate were consumed every 3 h. One of the caveats of this investigation was the very low total dose of protein consumed. Eighty grams of protein over a 12-h period would be grossly inadequate for athletes performing high volumes of training as well as those who are extremely heavy (e.g., American football players, sumo wrestlers, field athletes, etc.). A follow-up study one year later from the same research group determined myofibrillar protein synthesis rates after randomizing participants into three different protein ingestion patterns and examined how altering the pattern of protein administration affected protein synthesis rates after a bout of resistance exercise [150]. Two key outcomes were identified. First, rates of myofibrillar protein synthesis rates increased in all three groups. Second, when four, 20-g doses of whey protein isolate were consumed every 3 h over a 12-h post-exercise period, significantly greater (in comparison to the other two patterns of protein ingestion) rates of myofibrillar protein synthesis occurred. In combining the results of both studies, one can conclude that ingestion of intermediate protein doses (20 g) consumed every 3 h creates more favorable changes in both whole-body as well as myofibrillar protein synthesis [149, 150]. Although both studies employed short-term methodology and other patterns or doses have yet to be examined, the results thus far consistently suggest that the timing or pattern in which high-quality protein is ingested may favorably impact net protein balance as well as rates of myofibrillar protein synthesis.

An important caveat to these findings is that supplementation (in most cases) was provided in exclusion of other macronutrients over the duration of the study. Consumption of mixed meals delays gastric emptying and thus may result in different metabolic effects. Moreover, the fact that whey is a fast-absorbing protein source [151] further confounds the ability to generalize results to traditional mixed-meal diets, as the potential for oxidation is increased with larger dosages, particularly in the absence of other macronutrients. Whether acute MPS responses translate to longitudinal changes in hypertrophy or fiber composition also remains to be determined [120]. In addition to these aforementioned studies, extensive work by Arciero and colleagues has directly examined the combined effect of meal frequency and timing along with the distribution of protein intake with [129, 130, 152–156] and without [157, 158] exercise training in both short-term (3 months) and longer-term (> 1 year) interventions using a “protein pacing” model. Protein pacing involves the consumption of 20–40 g servings of high-quality protein, from both whole food and protein supplementation, evenly spaced throughout the day, approximately every 3 h. The first meal is consumed within 60 min of waking in the morning, and the last meal is eaten within 3 h of going to sleep at night. Arciero and colleagues [129, 130] have most recently demonstrated increased muscular strength and power in exercise-trained physically fit men and women using protein pacing compared to ingestion of similar sized meals at similar times but different protein contents, both of which included the same multi-component exercise training during a 12-week intervention.

In this respect and in consideration of alterations in the time between protein feedings, one must also consider the impact of the “muscle full” effect introduced by Millward et al. [159] and later refined by Atherton et al. [160] where it was speculated that a sensing mechanism was present in muscle that governed overall rates of muscle protein growth. In support of this theory one can point to the well characterized changes seen in peak MPS rates within 90 min after oral ingestion of protein [160] and the return of MPS rates to baseline levels in approximately 90 min despite elevations in serum amino acid levels [161]. Thus if efficacious protein feedings are placed too close together it remains possible that the ability of skeletal muscle anabolism to be fully activated might be limited. While no clear consensus exists as to the acceptance of this theory, conflicting findings exist between longitudinal studies that did provide protein feedings in close proximity to each other [16, 110, 153], making this an area that requires more investigation. Finally, while the mechanistic implications of pulsed vs. bolus protein feedings and their effect on MPS rates may help ultimately guide application, the practical importance has yet to be demonstrated.

### Pre-sleep protein intake

Eating before sleep has long been controversial [162–164]. However, methodological considerations in the original studies such as the population used, time of feeding, and size of the pre-sleep meal confounds any conclusions that can be drawn. Recent work using protein-centric beverages consumed 30-min before sleep and 2 h after the last meal (dinner) have identified pre-sleep protein consumption as advantageous to MPS, muscle recovery, and overall metabolism in both acute and long-term studies [165, 166]. For example, data indicate that 30–40 g of casein protein ingested 30-min prior to sleep [167] or via nasogastric tubing [168] increased overnight MPS in both young and old men, respectively.

Likewise, in an acute setting, 30 g of whey protein, 30 g of casein protein, and 33 g of carbohydrate consumption 30-min pre-sleep resulted in elevated morning resting metabolic rate in fit young men compared to a non-caloric placebo [169]. Similarly, although not statistically significant, morning increases in resting metabolic rate were reported in young overweight and/or obese women [170]. Of particular interest is that Madzima et al. [169] reported that the respiratory quotient (RQ) the morning after pre-sleep nutrient intake was similar for the placebo and casein protein trials, while both carbohydrate and whey protein producedincreasedRQ compared to placebo. This infers that casein protein consumed pre-sleep maintains overnight lipolysis and fat oxidation. This finding was verifiedwhen Kinsey et al. [171] designed a study using the microdialysis technique to measure interstitial glycerol concentrations overnight from the subcutaneous abdominal adipose tissue following 30 g of casein or a flavor and sensory-matched noncaloric placebo in obese men. It was concluded that pre-sleep casein did not blunt overnight lipolysis or fat oxidation. Similar to Madzima et al. [169] who compared pre-sleep ingestion of either casein or carbohydrate, Kinsey et al. [171] also concluded that pre-sleep casein did not result in elevated insulin the next morning along with decreased ratings of hunger in an overweight population. Of note, it appears that previous exercise training completely ameliorates any rise in insulin when eating at night before sleep [172] and the combination of pre-sleep protein and exercise has been shown to reduce blood pressure and arterial stiffness in young obese women with prehypertension and hypertension [173].

To date, only two studies involving nighttime protein have been carried out for longer than four weeks. Snijders et al. [174], randomly assigned young men (22 ± 1 years old) to consume a protein-centric supplement (27.5 g of casein protein, 15 g of carbohydrate, and 0.1-g of fat) or a noncaloric placebo every night before sleep while also completing a 12-week progressive resistance exercise training program (3 times per week). The group receiving the protein-centric supplement each night before sleep had greater improvements in muscle mass and strength over the 12-weeks. Of note, this study was non-nitrogen balanced and the protein group received approximately 1.9 g/kg/day of protein compared to 1.3 g/kg/day in the placebo group. More recently, in a nitrogen-balanced design using young healthy men and women, Antonio et al. [175] supplemented participants with 54 g of casein protein for eight weeks either in the morning (any time before 12 pm) or in the evening (90 min or less before sleep) and compared changes in body composition, strength performance outcomes. All subjects maintained their usual exercise program. The authors reported no differences in body composition or performance between the morning and evening casein supplementation groups. A potential explanation for the lack of findings might stem from the already high intake of protein by the study participants before the study commenced. However, it is worth noting that although not statistically significant, the morning group added 0.4 kg of lean body mass compared to 1.2 kg in the evening group even though the habitual diet of the trained subjects in this study was reported to be 1.7 to 1.9 g/kg/day of protein. Thus, it appears that protein consumption in the evening before sleep represents another opportunity to consume protein and other nutrients. Certainly more research is needed to determine if timing per se, or the mere addition of total daily protein can affect body composition or recovery via nighttime feeding.

## Conclusions

Nutrient timing is an area of research that continues to gather interest from researchers, coaches, and consumers. In reviewing the literature, two key considerations should be made. First, all findings surrounding nutrient timing require appropriate context because factors such as age, sex, fitness level, previous fueling status, dietary status, training volume, training intensity, program design, and time before the next training bout or competition can influence the extent to which timing may play a role in the adaptive response to exercise. Second, nearly all research within this topic requires further investigation. The reader must keep in perspective that in its simplest form nutrient timing is a feeding strategy that in nearly all situations may be helpful towards the promotion of recovery and adaptations towards training. This context is important because many nutrient timing studies demonstrate favorable changes that do not meet statistical thresholds of significance thereby leaving the reader to interpret the level of practical significance that exists from the findings. In this respect, it is the position of the ISSN that when a strategy may either help or have a neutral effect and fits within that individual’s daily schedule and ability to comply, then from a purely practical perspective, it is worth employing. It is noteworthy that differences in real-world athletic performances can be so small that even strategies that offer a modicum of benefit are still worth pursuing. One must remember that the overarching purpose of any nutritional strategy is to enhance the adaptive response to acute and/or chronic exercise. In nearly all such situations, this approach results in an athlete receiving a combination of nutrients at specific times that may be helpful and has not yet shown to be harmful. This perspective also has the added advantage of offering more flexibility to the fueling considerations a coach or athlete may employ. Using this approach, when both situations (timed or non-timed ingestion of nutrients) offer positive outcomes then our perspective is to advise an athlete to follow whatever strategy offers the most convenience or compliance if for no other reason than to deliver vital nutrients in amounts at a time that will support the physiological response to exercise.

Finally, it is advisable to remind the reader that due to the complexity, cost and invasiveness required to answer some of these fundamental questions, research studies often employ small numbers of study participants. Also, for the most part studies have primarily evaluated men. This latter point is particularly important as researchers have documented that females oxidize more fat when compared to men, and also seem to utilize endogenous fuel sources to different degrees [28–30]. Furthermore, the size of potential effects tends to be small, and when small potential effects are combined with small numbers of study participants, the ability to determine statistical significance remains low. Nonetheless, this consideration remains relevant because it underscores the need for more research to better understand the possibility of the group and individual changes that can be expected when the timing of nutrients is manipulated.

### Practical applications

- In many situations, the efficacy of nutrient timing is inherently tied to the concept of optimal fueling. Thus, the importance of adequate energy, carbohydrate, and protein intake must be emphasized to ensure athletes are properly fueled for optimal performance as well as to maximize potential adaptations to exercise training.
- Prolonged exercise (> 60 – 90 min) of moderate to high intensity (65–80% VO_2_max) relies heavily upon endogenous carbohydrate stores, and timing strategies to maximize these stores (carbohydrate loading or glycogen supercompensation strategies) have been shown to facilitate recovery and offset these changes.
- High-intensity exercise (particularly in hot and humid conditions) demands aggressive carbohydrate and fluid replacement. Consumption of 1.5–2 cups (12–16 fluid ounces) of a 6–8% carbohydrate solution (6–8 g carbohydrate per 100 mL of fluid) has been shown as an effective strategy to replace fluid, sustain blood glucose levels and promote performance. The need for carbohydrate replacement increases in importance as training and competition extend beyond 70 min of activity and the need for carbohydrate during shorter durations is less established.
- Rapid ingestion of high amounts of carbohydrates (≥ 1.2 g/kg/h) for four to 6 h soon after exhausting exercise can rapidly stimulate replenishment of muscle glycogen.
- Adding protein (0.2–0.5 g/kg/h) to carbohydrate increases the rate of glycogen resynthesis when ingesting < 1.2 g/kg/h of carbohydrate. Moreover, the additional protein may minimize muscle damage, promote favorable hormone balance and accelerate recovery from intense exercise.
- For athletes completing high volumes (i.e., ≥ 8 h) of exercise per week and subsequently requiring the need to continually and rapidly replenish endogenous glycogen stores, the single most effective strategy to maximize endogenous glycogen stores is the consumption of a daily diet high in carbohydrate (8–12 g/kg/day).
- The use of a 20–40-g dose of a high-quality protein source that contains approximately 10–12 g of the EAA maximizes MPS rates that remain elevated for three to four hours following exercise.
- Protein consumption during the peri-workout period is a pragmatic and sensible strategy for athletes, particularly those who perform high volumes of exercise. Not consuming protein post-workout (e.g., waiting for several hours post-exercise) offers no benefits.
- The impact of delivering a dose of protein (with or without carbohydrates) during the peri-workout period over the course of several weeks may operate as a strategy to heighten adaptations to exercise. Key factors that may influence the overall outcomes include one’s total daily protein intake, an individual’s training status and when their last dose of protein was consumed.
- Like carbohydrate, timing related considerations for protein appear to be of lower priority than the ingestion of optimal amounts of daily protein (1.4–2.0 g/kg/day).
- In the face of restricting caloric intake for weight loss, altering meal frequency has shown limited effects on body composition. However, more frequent meals may be more beneficial when accompanied by an exercise program. The impact of altering meal frequency in combination with an exercise program in non-athlete or athlete populations warrants further investigation. It is established that altering meal frequency (outside of an exercise program) may help with controlling hunger, appetite and satiety.
- Nutrient timing strategies that involve changing the distribution of intermediate-sized protein doses (20–40 g or 0.25–0.40 g/kg/dose) every three to 4 h best supports increased MPS rates across the day and favorably enhances body composition and physical performance outcomes. One must also consider that other factors such as the type of exercise stimulus, training status, and consumption of mixed macronutrient meals versus sole protein feedings can all impact how protein is metabolized across the day.
- When consumed within 30 min before sleep, 30–40 g of casein may increase MPS rates and improve strength and muscle hypertrophy. In addition, protein ingestion prior to sleep may increase morning metabolic rate while exerting minimal influence over lipolysis rates. In addition, pre-sleep protein intake can operate as an effective way to meet daily protein needs while also providing a metabolic stimulus for muscle adaptation.
- Altering the timing of energy intake (i.e., total calories over the course of a day) may improve weight loss, body composition changes, and health-related markers, particularly when a greater proportion of calories are consumed during breakfast and to a greater extent when this meal provides higher amounts of dietary protein.
